# Serum *N*-Glycan Profiling Predicts Prognosis in Patients Undergoing Hemodialysis

**DOI:** 10.1155/2013/268407

**Published:** 2013-12-23

**Authors:** Shingo Hatakeyama, Maho Amano, Yuki Tobisawa, Tohru Yoneyama, Megumi Tsushima, Kazuko Hirose, Takahiro Yoneyama, Yasuhiro Hashimoto, Takuya Koie, Hisao Saitoh, Kanemitsu Yamaya, Tomihisa Funyu, Shin-Ichiro Nishimura, Chikara Ohyama

**Affiliations:** ^1^Department of Urology, Hirosaki University Graduate School of Medicine, Hirosaki 036-8562, Japan; ^2^Faculty of Advanced Life Science and Frontier Research Center for Post-Genome Science and Technology, Hokkaido University, Sapporo 001-0021, Japan; ^3^Department of Advanced Transplant and Regenerative Medicine, Hirosaki University Graduate School of Medicine, Hirosaki 036-8562, Japan; ^4^Department of Radiological Technology, Hirosaki University School of Health Sciences, Hirosaki 036-8562, Japan; ^5^Department of Urology, Oyokyo Kidney Research Institute, Hirosaki 036-8243, Japan

## Abstract

*Background*. The aim of this study is to evaluate the usefulness of serum *N*-glycan profiling for prognosis in hemodialysis patients. *Methods*. Serum *N*-glycan analysis was performed in 100 hemodialysis patients in June 2008 using the glycoblotting method, which allows high-throughput, comprehensive, and quantitative *N*-glycan analysis. All patients were longitudinally followed up for 5 years. To evaluate the independent predictors for prognosis, patients' background, blood biochemistry, and *N*-glycans intensity were analyzed using Cox regression multivariate analysis. Selected *N*-glycans and independent factors were evaluated using the log-rank test with the Kaplan-Meier method to identify the predictive indicators for prognosis. Each patient was categorized according to the number of risk factors to evaluate the predictive potential of the risk criteria for prognosis. *Results*. In total, 56 *N*-glycan types were identified in the hemodialysis patients. Cox regression multivariate analysis showed cardiovascular events, body mass index, maximum intima media thickness, and the serum *N*-glycan intensity of peak number 49 were predictive indicators for overall survival. Risk classification according to the number of independent risk factors revealed significantly poor survival by increasing the number of risk factors. *Conclusions*. Serum *N*-glycan profiling may have a potential to predict prognosis in patients undergoing hemodialysis.

## 1. Introduction

Patients with end-stage renal disease that require hemodialysis are at extremely high risk for cardiovascular (CV) disease and mortality [1, 2]. Their risk for CV disease is 10- to 20-fold higher than that for healthy individuals in the age- and gender-matched general population [3]. In addition to conventional prognostic risk factors, such as age, hypertension (HTN), and diabetes mellitus (DM), other potential risk factors exist, including anemia, endothelial dysfunction [4], oxidant stress [5], chronic inflammation (C-reactive protein and cytokines) [6], vascular stiffness, and vascular calcification [7, 8]. Although several risk factors have been reported, the clinical efficacy of these biomarkers for the prediction of prognosis is limited and unsatisfactory. New approaches to identify the risk factors for prognosis are needed to overcome this problem.

Glycosylation is known to play an important role in various biological functions. The role of glycosylation in cancer has been well examined; tumor malignancy is closely associated with the type of glycan expressed in bladder cancer [9], germ cell tumors [10], prostate cancer [11], colorectal cancer [12], hepatocellular cancer [13], and pancreatic cancer [14]. However, evaluation of serum *N*-glycans as a marker for predictor of prognosis has not been attempted in hemodialysis patients. In this study, serum *N*-glycan profiling was evaluated as a predictor of prognosis in hemodialysis patients. The glycoblotting method was utilized, which allows high-throughput, comprehensive, and quantitative *N*-glycan analysis.

## 2. Materials and Methods

This study was performed in accordance with the ethical standards of the Declaration of Helsinki and approved by the ethical committee of the Oyokyo Kidney Research Institute. Written informed consent was obtained from all the serum donors.

In June 2008, 530 patients receiving maintenance hemodialysis were treated at the Oyokyo Kidney Research Institute, Hirosaki, Japan. Of these, informed consent was obtained from 100 patients (mean age, 65 ± 7.0 years) who were enrolled in this study. Patients were included if the following criteria were met: (1) age, 20–80 years; (2) expected survival, >6 months; (3) provision of written informed consent to participate; (4) Eastern Cooperative Oncology Group performance status (ECOG-PS) score [15], 0 (fully active, able to carry on all predisease performance without restriction) or 1 (restricted in physically strenuous activity but ambulatory and able to carry out work of a light or sedentary nature); (5) baseline abdominal computed tomography (CT) had been performed within 12 months prior to enrollment; and (6) no malignancies. Patients were excluded if they had any other concomitant malignant disease or medical condition likely to result in death within 6 months of study enrollment. Serum samples were collected before the maintenance hemodialysis session in June 2008 and stored at −80°C.

### 2.1. Abdominal CT and Evaluation of Aortic Calcification Index (ACI)

ACI was quantitatively measured on abdominal CT (TSX-021B, Toshiba Medical systems Corp., Ohtawara, Japan) images above the bifurcation of the common iliac artery scanned 10 times at 10 mm intervals, as previously described [8].

### 2.2. Measurement of Maximum Intima Media Thickness (max-IMT)

We measured the thickness of the intima media complex on the far wall of the bilateral common carotid artery using high resolution B-mode ultrasonography (SSA-700A; Toshiba, Tokyo) with a 12.0 MHz linear-type transducer and defined the maximum value including plaques as IMT [8]. IMT was evaluated at the start of this study.

### 2.3. Patients' Data Collection

Patient data collected in June 2008 included age, gender, duration of dialysis, presence of DM, ACI, max-IMT, mean systolic and diastolic blood pressure (BP) at sample collection, date of death or last followup, and results of blood biochemical analysis such as hemoglobin (Hb), serum albumin (Alb), serum corrected calcium (Ca), serum phosphorus (P), and C-reactive protein (CRP) levels. All the patients were longitudinally followed up during the standard maintenance hemodialysis for 5 years or until death. The mean observation times for the hemodialysis patients after dialysis and sample collection were 90 ± 57 and 34 ± 10 months, respectively.

### 2.4. Glycoblotting Method and Mass Spectrometry Analysis

**Figure 1:**
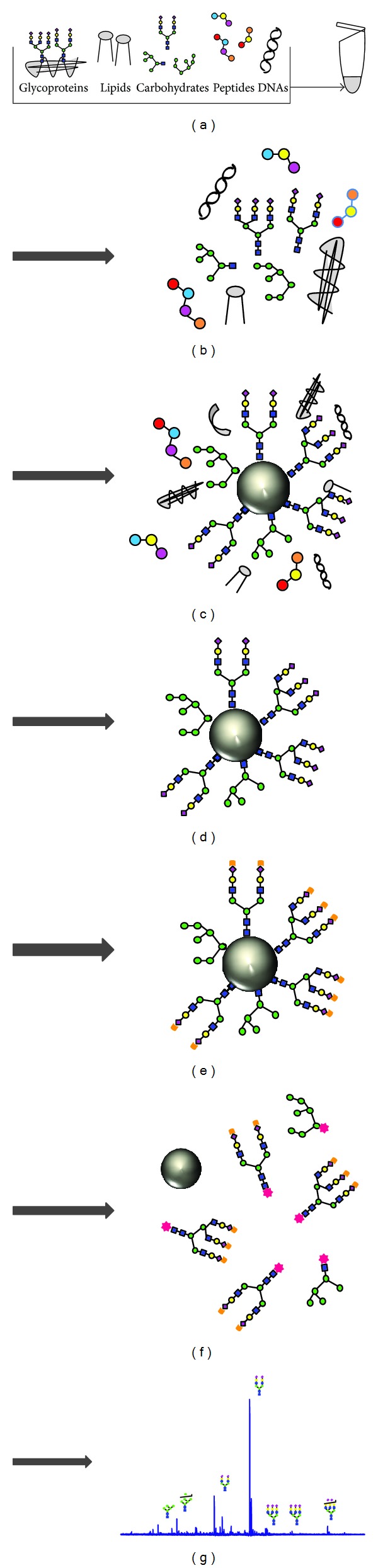
General protocol for the integrated glycoblotting technique and workflow for glycoblotting-based high-throughput clinical glycan analysis. Serum samples of 10 *μ*L (a) were applied to the “Sweet Blot” automated machine for glycoblotting. After enzymatic cleaving from serum protein, the total serum *N*-glycans released in the digest mixture (b) were directly mixed with BlotGlyco H beads (Sumitomo Bakelite, Co., Tokyo, Japan) to capture *N*-glycans (c). After the beads had been separated from other molecules by washing (d), sialic acid was methyl esterified (e). The processed *N*-glycans were then labeled with benzyloxyamine (BOA) and released from the BlotGlyco H beads (f). Mass spectra of BOA-labeled *N*-glycans were acquired with an Ultraflex III instrument (Bruker Daltonics, Germany) (g).

All serum samples from the 100 patients undergoing maintenance hemodialysis were collected before the last hemodialysis session in June 2008. Serum *N*-glycan analysis was performed according to the procedure described in a previous study [16]. Briefly, the general steps were as follows: (1) enzymatic cleaving from serum protein, (2) chemoselective capture of reducing sugars onto hydrazide-functionalized beads using the glycoblotting technology, (3) washing to remove impurities, (4) on-bead methyl esterification of the sialic acid residues followed by reduction of the hydrazone linkage, (5) recovery of modified *N*-glycans by reduction of the disulfide bond, (6) mass spectrometry, and (7) data analysis (Figure 1).

**Figure 2:**
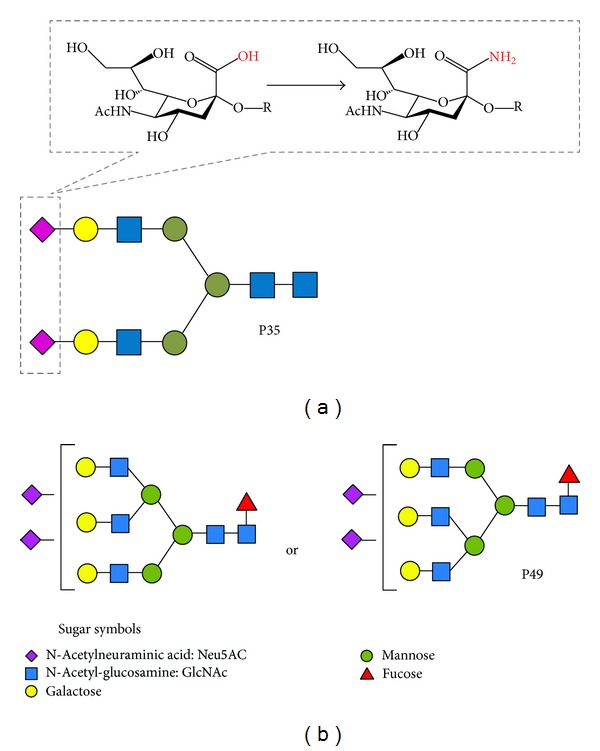
*N*-Glycan symbols of internal standard and significant *N*-glycans selected by logistic regression analysis. *O*-Benzyl hydroxylamine hydrochloride (BOA)-labeled amidated sialic acids (A2 amide), which do not exist in nature (peak number 35), were used as the internal standard (a). Putative structures of significant *N*-glycans selected using Cox regression analysis are presented. Peak number 49 (P49) was selected for overall survival (b).

*O*-Benzyl hydroxylamine hydrochloride-labeled amidated sialic acid (*m*/*z*; 2348.8619) was used as the internal standard. Its structure is presented in Figure 2(a). Fifty-six *N*-glycans (Table 1) were selected from the matrix-assisted laser desorption/ionization-time of flight (MALDI-TOF) spectra peaks using FlexAnalysis version 3 (Bruker Daltonics, Bremen, Germany). The area of the isotopic peaks of each glycan was normalized to the internal standard. The compositions and structures of the glycans were suggested by the GlycoMod Tool (http://br.expasy.org/tools/glycomod/) and GlycoSuite DB (http://glycosuitedb.expasy.org/glycosuite/glycodb). All the statistical analyses were performed using Matlab version 7.4 (Math works, Inc., Natick, MA, USA) with the Statistics Toolbox.

### 2.5. Statistical Analysis

Statistical analyses of the clinical data were performed using SPSS ver. 19.0 (SPSS, Inc., Chicago, IL, USA) and GraphPad Prism 5.03 (GraphPad Software, San Diego, CA, USA). Categorical variables were compared using Fisher's exact test. The differences between deceased patients and survivors were statistically compared by Student's *t*-test for normal distribution model or Mann-Whitney *U* test for nonnormal distribution model. *P* values < 0.05 were considered statistically significant.

Age, gender, hemodialysis duration, presence of DM, ACI, maxi-IMT, BP, Hb, Alb, Ca, P, CRP, and *N*-glycans intensity were analyzed using uni- and multivariate Cox regression analysis to evaluate the independent predictors for prognosis. A receiver operating characteristic (ROC) curve was used to determine the cutoff value of continuous variables for prognosis. Optimal cutoff points were calculated by the following formula [17]: (1 − sensitivity)^2^ + (1 − specificity)^2^. Selected *N*-glycans were evaluated using the log-rank test with the Kaplan-Meier method to identify their potential as predictors of prognosis.

Each patient was categorized according to the number of selected risk factors using Cox regression multivariate analysis to evaluate the predictive potential of risk criteria for prognosis using the log-rank test with the Kaplan-Meier method. Each existing risk factor was scored as 1 risk, and scores for all the risk factors were added together. Patients were classified into 3 groups according to the number of risk factors: the low-risk group (patients with no risk factors), the intermediate-risk group (1 or 2 risk factors), and the high-risk group (3 or 4 risk factors).

## 3. Results

In total, 56 types of *N*-glycans were identified from hemodialysis patients by glycoblotting methods (Table 1).  Table 2 summarizes the demographic characteristics of the study cohort. The mean age was 65 ± 7.0 years, and the median followup period was 60 months (range, 4.9–60). Presence of DM, BMI, CRP, max-IMT, and the serum *N*-glycan intensity of peak number 49 (P49) were significantly different between deceased and survived patients.

**Figure 3:**
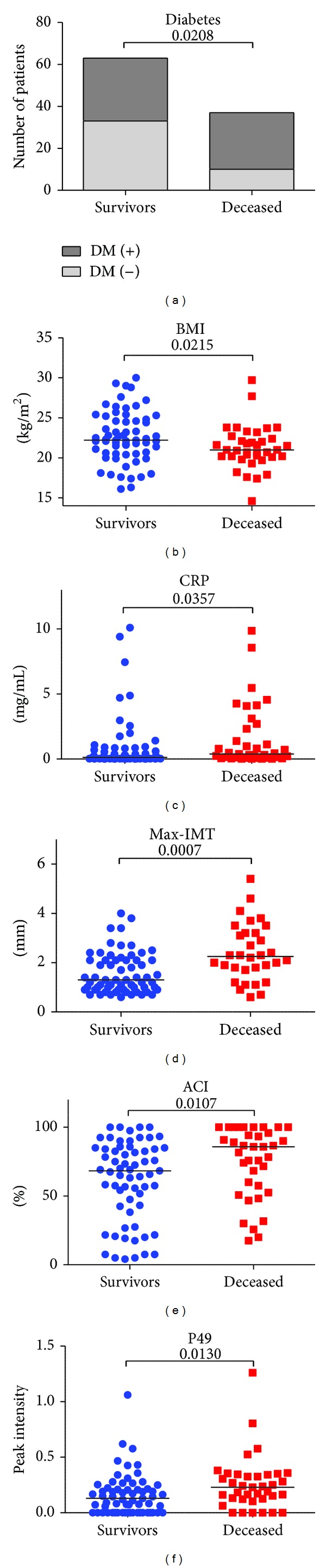
Patients' parameters significantly associated with overall survival. Selected parameters for overall survival were presence of DM (a), BMI (b), CRP (c), max-IMT (d), ACI (e), and intensity of P49 (f).

**Figure 4:**
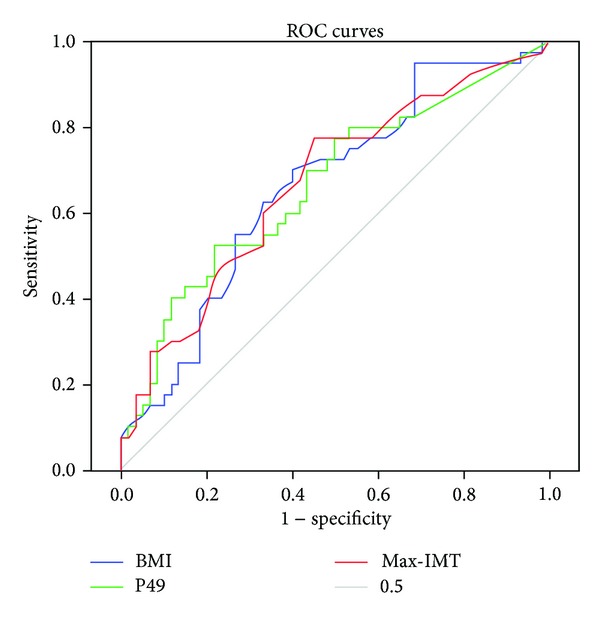
Receiver operating characteristic (ROC) curves. Areas under the curves (AUCs) in BMI, max-IMT, and intensity of P49 were 0.66, 0.67, and 0.67, respectively.

**Figure 5:**
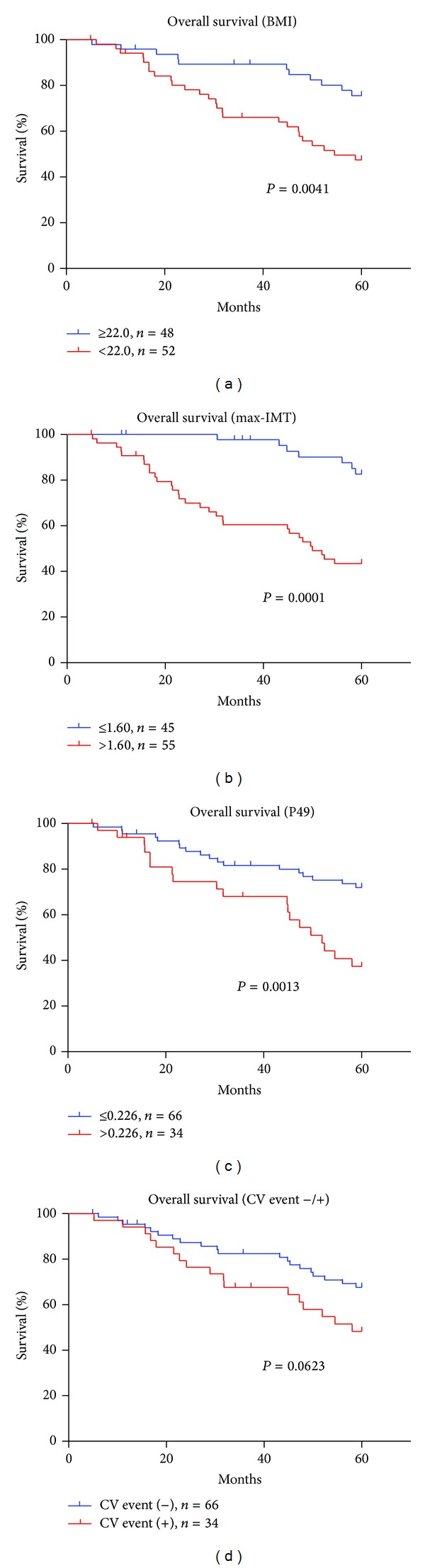
Overall survival from the cutoff points. Significantly poor overall survival was observed in patients with BMI < 22.0 (a), max-IMT > 1.6 (b), and P49 > 0.226 (c) using the log-rank test and the Kaplan-Meier method. Overall survival in patients with cardiovascular events showed not significant but boundary effects on prognosis (*P* = 0.0623) (d).

To evaluate the independent predictors for prognosis, patients' profiles including age, gender, dialysis vintage, presence of DM, BMI, BP, Hb, Alb, Ca, P, CRP, max-IMT, ACI, CV events, and the serum *N*-glycan intensity of P49 were analyzed using the Cox regression uni- and multivariate analysis (Table 3). The univariate analysis for prognosis revealed that presence of DM, BMI, Alb, CRP, max-IMT, ACI, CV events, and the serum *N*-glycan intensity of P49 were potential predictors (Figures 3(a), 3(b), 3(c), 3(d), 3(e), and 3(f), resp.). The *N*-glycan symbols of P49 are presented in Figure 2(b). The multivariate analysis selected BMI (HR = 0.83), max-IMT (HR = 1.49), intensity of P49 (HR = 8.39), and presence of CV events (HR = 2.88). The ROC analysis showed area under the curve (AUC) for BMI, max-IMT, and P49 was 0.66 (*P* = 0.006), 0.67 (*P* = 0.005), and 0.67 (*P* = 0.004), respectively (Figure 4). The optimal cutoff values for BMI, max-IMT, and P20 for overall survival were 22.0, 1.6, and 0.226, respectively (Table 4). The log-rank test and Kaplan-Meier method indicated significantly poor survival in patients with BMI < 22.0 (*P* = 0.0041), max-IMT > 1.6 (*P* < 0.001), and the intensity of P49 > 0.226 (*P* = 0.013) (Figures 5(a), 5(b), and 5(c), resp.) compared with those in other patients. The patients with CV event showed boundary effects on prognosis (*P* = 0.0623) (Figure 5(d)).

**Figure 6:**
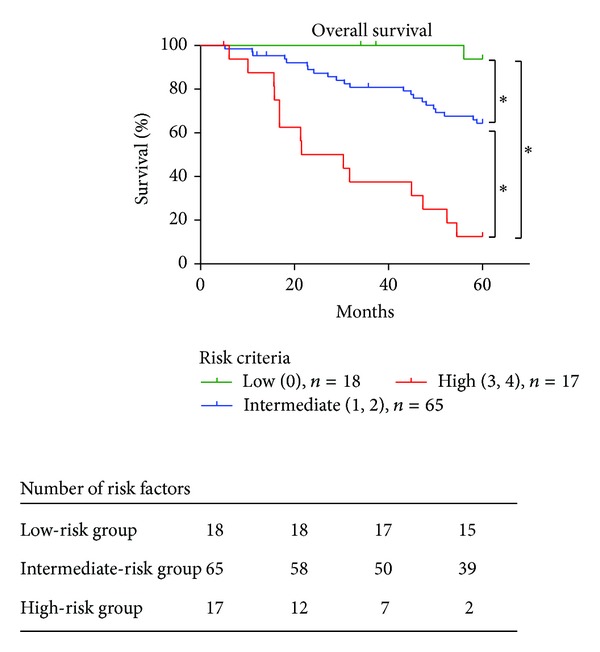
Risk classification according to the number of independent risk factors for overall survival. Patients were categorized into 3 groups according to the number of independent risk factors for overall survival: the low-risk group (patients with no risk factors), the intermediate-risk group (1 or 2 risk factors), and the high-risk group (3 or 4 risk factors). Risk classification revealed significantly poor prognosis by increasing the number of risk factors (*P < 0.001). The patients with high-risk group showed significantly poor prognosis compared with those in the other groups.

Patients were categorized according to the number of independent risk factors for overall survival. Patients were classified into 3 groups: the low-risk group (patients with no risk factors), the intermediate-risk group (1 or 2 risk factors), and the high-risk group (3 or 4 risk factors). Risk classification revealed significantly poor prognosis by increasing the number of risk factors. The patients with high-risk group showed significantly poor prognosis compared with those in the other groups (Figure 6). The estimated 5-year survival rates were 94%, 64%, and 13%, respectively.

## 4. Discussion

Large-scale quantitative glycomics is an important and promising discipline. Differences in glycan expression between the diseased and healthy states may be useful for the diagnosis or prognosis of diseases [18, 19]. Several reports on serum glycan biomarkers have distinguished patients with breast and stomach cancer from healthy subjects [20, 21]. However, no polymerase chain reaction-like glycan amplification technology is available for glycans because the glycan biosynthetic process is not template-driven and is subject to multiple sequential and competitive enzymatic steps. Although partial peptide fragments detected by proteomics are fully supported by full-length protein/DNA sequence databases, glycan analysis requires enrichment of all glycans from highly complicated mixtures, such as serum, cells, and tissues. Therefore, the crucial bottleneck for structural and functional glycan analysis is a tedious and time-consuming multistep process to purify trace amounts of glycans.

In the present study, the recently established technology of high-throughput quantitative glycan analysis by glycoblotting methods was used for analysis of serum *N*-glycans in patients with end-stage renal disease. To our knowledge, this is the first analysis to identify serum *N*-glycans as a biomarker in hemodialysis patients. Our results suggest the potential clinical value of quantitative analysis of whole-serum *N*-glycan profiles acquired using a MALDI-TOF mass spectrometer. The spectrometric intensity of peak number 49 was identified as statistically significant. A significant difference was observed in survival between these optimal cutoff points. Recently, the diagnostic utility of serum *N*-glycans in pancreatic cancer was reported using the glycoblotting method, which allows high-throughput, comprehensive, and quantitative *N*-glycan analysis [19]. Their results suggested that the use of the glycoblotting method may provide new knowledge regarding prognosis and therapeutic strategies for improvement of prognosis in hemodialysis patients.

Although serum *N*-glycan expression was revealed as a useful prognostic marker in hemodialysis patients in this study, it has several limitations. First, only a small number of patients were included, and risk factors such as dyslipidemia and smoking history were not included. Second, information regarding the carrier proteins of *N*-glycans and the interpretation of these changes of *N*-glycans was not available for the hemodialysis patients included in this study. In addition, the origin of the *N*-glycans alterations observed in this study was not identified. Our preliminary data regarding carrier proteins of the altered carbohydrates suggested that immunoglobulin mediated by systemic immune response may play a critical role in serum glycan changes (data not shown). Future studies should determine whether these alterations directly result from cardiovascular damage or from biological reactions. A third limitation of this study is that the longitudinal patterns of changes in *N*-glycans from early-stage to end-stage renal disease were not investigated. To understand the patterns of glycan changes, comparison with healthy subjects is necessary. The usefulness of the glycoblotting method for early detection of life-threatening chronic renal disease may have a great impact on preventing renal failure-associated mortality in the future.

Despite these limitations, alterations in *N*-glycan expression were clearly demonstrated as potential markers for the prediction of cardiovascular events and prognosis in this study. The integrated glycoblotting technique used here may greatly facilitate research for new biomarkers in hemodialysis patients. Future large-scale prospective studies may determine the clinical significance of these carbohydrate biomarkers.

## 5. Conclusion

Serum* N*-glycan analysis may have the potential to predict prognosis in patients undergoing hemodialysis. Glycoblotting may be a promising and useful method for discovery of new prognostic biomarkers. However, the novel *N*-glycan needs to be analyzed using a larger cohort prior to clinical utility and validity is established.

## Figures and Tables

**Table 1:** Human serum *N*-glycans targeted in this study.

Peak no.	*m*/*z*	Composition	Peak no.	*m*/*z*	Composition
1	1362.48	(Hex)2 + (Man)3(GlcNAc)2	30	2260.8349	(Hex)1(HexNAc)3(dHex)1(NeuAc)1 + (Man)3(GlcNAc)2
2	1387.51	(HexNAc)1(dHex)1 + (Man)3(GlcNAc)2	31	2263.8345	(Hex)2(HexNAc)3(dHex)2 + (Man)3(GlcNAc)2
3	1403.51	(Hex)1(HexNAc)1 + (Man)3(GlcNAc)2	32	2276.8298	(Hex)2(HexNAc)3(NeuAc)1 + (Man)3(GlcNAc)2
4	1444.53	(HexNAc)2 + (Man)3(GlcNAc)2	33	2279.8294	(Hex)2(HexNAc)3(NeuAc)1 + (Man)3(GlcNAc)2
5	1524.53	(Hex)3 + (Man)3(GlcNAc)2	34	2336.8509	(Hex)3(HexNAc)4 + (Man)3(GlcNAc)2
6	1549.57	(Hex)1(HexNAc)1(dHex)1 + (Man)3(GlcNAc)2	**35**	**2348.8619**	**Internal standard (BOA-labeled A2-amide)**
7	1590.59	(HexNAc)2(dHex)1 + (Man)3(GlcNAc)2	36	2378.8615	(Hex)2(HexNAc)2(NeuAc)2 + (Man)3(GlcNAc)2
8	1606.59	(Hex)1(HexNAc)2 + (Man)3(GlcNAc)2	37	2406.8928	(Hex)1(HexNAc)3(dHex)2(NeuAc)1 + (Man)3(GlcNAc)2
9	1647.61	(HexNAc)3 + (Man)3(GlcNAc)2	38	2419.8932	(Hex)1(HexNAc)3(NeuAc)2 + (Man)3(GlcNAc)2
10	1686.59	(Hex)4 + (Man)3(GlcNAc)2	39	2422.8877	(Hex)2(HexNAc)3(dHex)1(NeuAc)1 + (Man)3(GlcNAc)2
11	1708.62	(Hex)1(HexNAc)1(NeuAc)1 + (Man)3(GlcNAc)2	40	2438.8826	(Hex)3(HexNAc)3(NeuAc)1 + (Man)3(GlcNAc)2
12	1752.64	(Hex)1(HexNAc)2(dHex)1 + (Man)3(GlcNAc)2	41	2482.9088	(Hex)3(HexNAc)4(dHex)1 + (Man)3(GlcNAc)2
13	1768.64	(Hex)2(HexNAc)2 + (Man)3(GlcNAc)2	42	2498.9037	(Hex)4(HexNAc)4 + (Man)3(GlcNAc)2
14	1793.67	(HexNAc)3(dHex)1 + (Man)3(GlcNAc)2	43	2524.9194	(Hex)2(HexNAc)2(dHex)1(NeuAc)2 + (Man)3(GlcNAc)2
15	1809.67	(Hex)1(HexNAc)3 + (Man)3(GlcNAc)2	44	2584.9405	(Hex)3(HexNAc)3(dHex)1(NeuAc)1 + (Man)3(GlcNAc)2
16	1848.64	(Hex)5 + (Man)3(GlcNAc)2	45	2727.9988	(Hex)2(HexNAc)3(dHex)1(NeuAc)2 + (Man)3(GlcNAc)2
17	1854.68	(Hex)1(HexNAc)1(dHex)1(NeuAc)1 + (Man)3(GlcNAc)2	46	2743.9937	(Hex)3(HexNAc)3(NeuAc)2 + (Man)3(GlcNAc)2
18	1870.67	(Hex)2(HexNAc)1(NeuAc)1 + (Man)3(GlcNAc)2	47	2785.025	(Hex)2(HexNAc)4(NeuAc)2 + (Man)3(GlcNAc)2
19	1914.7	(Hex)2(HexNAc)2(dHex)1 + (Man)3(GlcNAc)2	48	2804.0148	(Hex)4(HexNAc)4(NeuAc)1 + (Man)3(GlcNAc)2
20	1955.72	(Hex)1(HexNAc)3(dHex)1 + (Man)3(GlcNAc)2	49	2890.0516	(Hex)3(HexNAc)3(dHex)1(NeuAc)2 + (Man)3(GlcNAc)2
21	1971.72	(Hex)2(HexNAc)3 + (Man)3(GlcNAc)2	50	3049.1048	(Hex)3(HexNAc)3(NeuAc)3 + (Man)3(GlcNAc)2
22	2010.69	(Hex)6 + (Man)3(GlcNAc)2	51	3109.1259	(Hex)4(HexNAc)4(NeuAc)2 + (Man)3(GlcNAc)2
23	2032.72	(Hex)3(HexNac)1(NeuAc)1 + (Man)3(GlcNAc)2	52	3195.1627	(Hex)3(HexNAc)3(dHex)1(NeuAc)3 + (Man)3(GlcNAc)2
24	2057.76	(Hex)1(HexNAc)2(dHex)1(NeuAc)1 + (Man)3(GlcNAc)2	53	3354.616	(Hex)3(HexNAc)3(NeuAc)4 + (Man)3(GlcNAc)2
25	2060.76	(Hex)2(HexNAc)2(dHex)2 + (Man)3(GlcNAc)2	54	3414.237	(Hex)4(HexNAc)4(NeuAc)3 + (Man)3(GlcNAc)2
26	2073.75	(Hex)2(HexNAc)2(NeuAc)1 + (Man)3(GlcNAc)2	55	3560.2949	(Hex)4(HexNAc)4(dHex)1(NeuAc)3 + (Man)3(GlcNAc)2
27	2114.78	(Hex)1(HexNAc)3(NeuAc)1 + (Man)3(GlcNAc)2	56	3719.3481	(Hex)4(HexNAc)4(NeuAc)4 + (Man)3(GlcNAc)2
28	2117.78	(Hex)2(HexNAc)3(dHex)1 + (Man)3(GlcNAc)2	57	3865.406	(Hex)4(HexNAc)4(dHex)1(NeuAc)4 + (Man)3(GlcNAc)2
29	2219.81	(Hex)2(HexNAc)2(dHex)1(NeuAc)1 + (Man)3(GlcNAc)2			

We identified 56 kinds of *N*-glycan types in the hemodialysis patients. Peak number 35 was an internal standard spiked for quantification. Compositional annotation and putative structures (presented as abbreviations) were collected using the GlycoSuite online database (Proteome Systems). Hex: hexose; HexNAc: *N*-acetyl hexosamine; dHex: deoxyhexose.

**Table 2:** Patient characteristics in the study groups.

	All	Survivors	Deceased	*P* value
*n*	100	63	37	* *
Age (years)	65 ± 7.0	64 ± 7.0	66 ± 7.0	*0.256*
Gender (M/F)	52/48	30/33	22/15	*0.252*
Dialysis vintage (months)	90 ± 57	87 ± 63	95 ± 48	*0.479*
Diabetes	57 (57%)	30 (48%)	27 (73%)	*0.013*
BMI (kg/m^2^)	22 ± 3.1	23 ± 3.2	21 ± 2.7	*0.022*
Blood pressure (mean, mmHg)	123 ± 16	124 ± 15	122 ± 17	*0.677*
Hemoglobin (g/dL)	9.9 ± 1.0	9.9 ± 0.9	10 ± 1.0	*0.800*
Albumin (g/dL)	3.7 ± 0.4	3.8 ± 0.3	3.6 ± 0.5	*0.059*
Correlated calcium (mg/dL)	9.4 ± 0.9	9.5 ± 0.9	9.3 ± 0.9	*0.275*
Phosphorus (mg/dL)	5.0 ± 1.3	5.2 ± 1.2	4.6 ± 1.4	*0.061*
C-reactive protein (mg/dL)	1.2 ± 2.2	0.9 ± 2.1	1.6 ± 2.4	*0.036*
max-IMT (mm)	1.9 ± 1.0	1.6 ± 0.8	2.4 ± 1.2	*0.001*
ACI (%)	66 ± 29	61 ± 29	75 ± 26	*0.011*
Cardiovascular events	34 (34%)	17 (17%)	17 (17%)	*1.000*
P49	0.20 ± 0.21	0.16 ± 0.19	0.26 ± 0.24	*0.013*

**Table 3:** Uni- and multivariate Cox regression analysis of biochemical markers and clinical factors associated with overall survival. HR: hazard ratio, CI: confidence interval.

Univariate analysis	*P* value	HR	95.0% CI	Multivariate analysis	*P* value	HR	95.0% CI
Age (years)	*0.191 *	1.03	0.99–1.08				
Gender (M/F)	*0.094 *	1.73	0.91–3.28				
Dialysis vintage (months)	*0.350 *	1.00	1.00-1.01				
Diabetes	*0.010 *	2.55	1.25–5.23				
BMI (kg/m^2^)	*0.009 *	0.87	0.78–0.97	BMI (kg/m^2^)	*0.004 *	0.83	0.73–0.94
Blood pressure (mean, mmHg)	*0.500 *	0.99	0.97–1.01				
Hemoglobin (g/dL)	*0.919 *	0.98	0.71–1.37				
Albumin (g/dL)	*0.001 *	0.24	0.11–0.54				
Correlated calcium (mg/dL)	*0.196 *	0.77	0.52–1.14				
Phosphorus (mg/dL)	*0.273 *	0.87	0.67–1.12				
C-reactive protein (mg/dL)	*0.013 *	1.16	1.03–1.30				
max-IMT (mm)	*0.000 *	1.66	1.25–2.19	max-IMT (mm)	*0.008 *	1.49	1.11–2.00
ACI (%)	*0.010 *	1.02	1.00–1.03				
Cardiovascular events	*0.003 *	2.61	1.39–4.91	Cardiovascular events	*0.002 *	2.88	1.46–5.69
P49	*0.000 *	11.40	3.45–37.3	P49	*0.000 *	8.39	2.64–26.7

**Table 4:** Receiver operating characteristic (ROC) curves for independent prognostic factors to determine the area under the curves (AUCs) and the optimal cutoff values.

Risk factor	AUC	*P* value	95% CI	Optimal cutoff value
BMI(kg/m^2^)	0.66	*0.006 *	0.55–0.77	22.0
max-IMT (mm)	0.67	*0.005 *	0.56–0.78	1.6
P49	0.67	*0.004 *	0.56–0.78	0.226

## Abbreviations

CV: Cardiovascular
ACI: Aortic calcification index
max-IMT: Maximum intima media thickness
BP: Blood pressure
HTN: Hypertension
DM: Diabetes mellitus
ECOG-PS: Eastern Cooperative Oncology Group performance status
Hb: Hemoglobin
Alb: Serum albumin
Ca: Serum corrected calcium
P: Serum phosphorus
CRP: C-reactive protein
P49: Peak number 49
ROC: Receiver operating characteristic
AUC: Area under the curve
MALDI-TOF: Matrix-assisted laser desorption/ionization-time of flight
BOA: Benzyloxyamine.
